# Echocardiographic Changes in Infants with Severe Congenital Diaphragmatic Hernia After Fetoscopic Endoluminal Tracheal Occlusion (FETO)

**DOI:** 10.1007/s00246-024-03735-y

**Published:** 2024-12-13

**Authors:** Catherine M. Avitabile, Sabrina Flohr, Leny Mathew, Yan Wang, Devon Ash, Juliana S. Gebb, Natalie E. Rintoul, Holly L. Hedrick

**Affiliations:** 1 Department of Pediatrics, University of Pennsylvania Perelman School of Medicine, Philadelphia, PA USA; 2 Division of Cardiology, Children’s Hospital of Philadelphia, 3401 Civic Center Boulevard, 8NW49, Philadelphia, PA 19104 USA; 3 Division of Pediatric General, Thoracic, and Fetal Surgery, Children’s Hospital of Philadelphia, Philadelphia, PA USA; 4 Richard D. Wood Center for Fetal Diagnosis and Treatment, Children’s Hospital of Philadelphia, Philadelphia, PA USA; 5 Division of Neonatology, Children’s Hospital of Philadelphia, Philadelphia, PA USA

**Keywords:** Congenital diaphragmatic hernia, Echocardiogram, Pulmonary hypertension, Strain

## Abstract

Fetoscopic endoluminal tracheal occlusion (FETO) induces lung growth and may improve survival in congenital diaphragmatic hernia (CDH) but the effect on post-natal right (RV) and left (LV) ventricular size and cardiac function is unknown. Quantitative measures of heart size and function including tricuspid annular plane systolic excursion Z-score (TAPSEZ), RV fractional area change (RVFAC), RV global longitudinal and free wall strain (RVGLS, RVFWS), RV/LV ratio, LV eccentricity index (LVEI), and LV M-mode diastolic and systolic Z-scores (LVIDDZ, LVIDSZ) were compared between FETO and control patients on first post-natal echocardiogram, prior to and post CDH repair, and on last available echocardiogram using non-parametric Wilcoxon rank-sum test in a single-center, retrospective cohort study. Linear regression models evaluated change over time, adjusting for clustering and interaction of echocardiogram parameters with time. Thirty-two patients (10 FETO, 22 control) met inclusion criteria. At first echocardiogram, FETO patients demonstrated lower RV/LV ratio and LVEI (p = 0.01 for both) indicating less RV dilation and less ventricular septal displacement, respectively. LV hypoplasia was less severe in FETO patients (p = 0.01 for both LVIDDZ and LVIDSZ) initially. After repair, FETO patients demonstrated better RV systolic function compared to control patients by FAC (p < 0.01), RVGLS (p = 0.02), and RVFWS (p = 0.05). Over time, FETO patients demonstrated greater improvements in RV/LV ratio and LVEI but smaller increases in LV dimensions compared to control patients. Improvements in RV function were similar between the groups. FETO patients demonstrate differences in cardiac size and function compared to control patients.

## Introduction

In congenital diaphragmatic hernia (CDH), herniation of abdominal organs into the thorax impairs normal development of the lung and pulmonary vasculature [1]. The triad of pulmonary hypoplasia, pulmonary hypertension (PH), and cardiac ventricular dysfunction is common. Despite improvement in survival over time, overall mortality remains > 25%, higher in patients with the most severe pulmonary hypoplasia [2]. As fetal lung growth is stimulated by tracheal occlusion, the fetoscopic endoluminal tracheal occlusion (FETO) procedure was developed for patients with moderate to severe CDH [3, 4]. The open-label randomized controlled Tracheal Occlusion to Accelerate Lung Growth (TOTAL) trial demonstrated higher survival to neonatal discharge in FETO patients with severe CDH compared to those of similar CDH severity randomized to standard postnatal care, although the incidence of preterm birth was higher in those in the FETO group [5]. In CDH generally, survival varies by category of cardiac ventricular dysfunction [6], however, the impact of FETO on postnatal right (RV) and left (LV) ventricular dysfunction is not known. Quantitative measures of RV and LV size and function by echocardiogram (echo) predict survival and inform treatment decisions regarding pulmonary vasodilators in other forms of pediatric PH [7, 8]. It is critical to describe ventricular function in CDH patients undergoing FETO in order to understand the effect of the treatment strategy on the heart and develop targeted treatment approaches.

Therefore, the objectives of this study were to compare quantitative echo measures of ventricular size, function, and PH in neonates with CDH who underwent FETO patients and control patients of similar CDH severity.

## Methods

We performed a single-center, retrospective cohort study at the Children’s Hospital of Philadelphia comparing echo findings in left CDH patients who underwent FETO and control patients eligible for the procedure but whose parents declined between September 1, 2016 and November 1, 2021. FETO was offered to families who met the following inclusion criteria: left CDH, intrathoracic liver, observed/expected lung to head ratio < 30%, mother’s age ≥ 18 years, singleton pregnancy, normal fetal karyotype, and gestational age at enrollment < 30 weeks. Patients were not considered for FETO due to maternal contra-indications to fetoscopic surgery, severe maternal medical conditions in pregnancy, technical limitations precluding fetoscopic surgery, preterm labor, or psychosocial concerns that could affect the procedure or post-procedure care. Survival to CDH surgical repair was required for inclusion in this echo study. Relevant demographic and clinical data were extracted from the medical record. All study procedures were approved by the Children’s Hospital of Philadelphia Institutional Review Board in accordance with the Declaration of Helsinki.

**Fig. 1:**
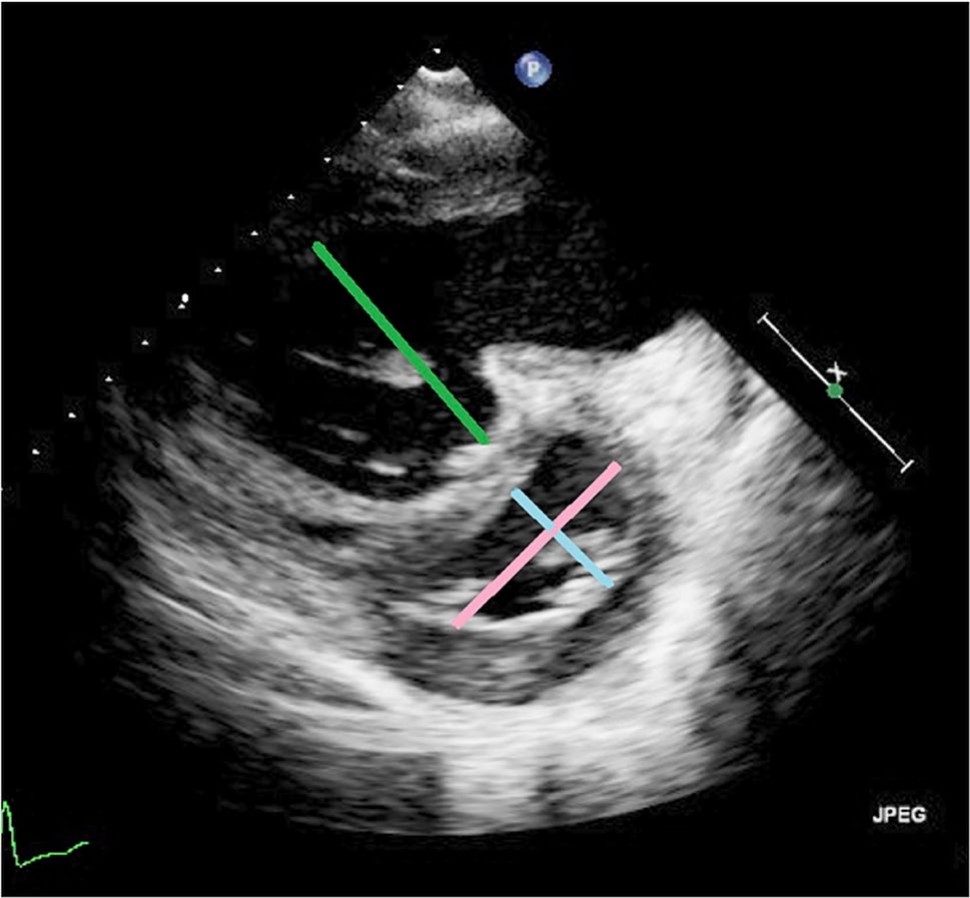
Measurement of LVEI and RV/LV ratio. End-systolic LVEI is measured as the ratio of the LV dimension parallel to the ventricular septum (pink) to the LV dimension perpendicular to the ventricular septum (blue) in parasternal short axis view. Systolic RV/LV ratio is measured as the ratio of the RV dimension (green) to the LV dimension perpendicular to the ventricular septum (blue) in the parasternal short axis view

Clinically indicated echocardiograms were performed using standard pediatric views with 3–8 MHz transducers on a Phillips IE33 machine (Phillips, Andover, MA, USA) in accordance with our echocardiography laboratory’s standard PH imaging protocol that was consistent over the study period and digitally stored in the Syngo Dynamics system (Siemens, USA). LV M-mode internal dimension Z-scores in diastole and systole (LVIDDZ, LVIDSZ) and LV shortening fraction were measured at the time of the study as per standard practice [9]. Available echocardiograms were retrospectively reviewed for additional analyses of ventricular function at four time points: baseline/first post-natal echo, closest echo pre-operative to CDH repair, closest echo post-operative from CDH repair, and last available echocardiogram prior to death or neonatal hospital discharge. Two pediatric cardiac sonographers (YW and DA), blinded to clinical characteristics, obtained offline measures of RV size and RV/LV systolic function that predict adverse outcomes in other forms of pediatric PH. RV and LV global longitudinal strain (RVGLS and LVGLS) and RV free wall strain (RVFWS) measurements of systolic myocardial deformation were obtained using Tomtec software (Image Arena 4.6; Munich, Germany). Strain is reported as a negative number, with greater absolute value indicating better systolic function [10]. Absolute value of the strain measures was used in this study [10]. RV systolic function was also assessed by tricuspid annular plane systolic excursion Z-scores (TAPSEZ) [11, 12] and RV fractional area change (RVFAC) calculated as the end-diastolic area minus the end-systolic area divided by the end-diastolic area [13]. Abnormal ventricular septal position was measured by end-systolic LV eccentricity index (LVEI), the ratio of the LV dimension parallel to the ventricular septum to the LV dimension perpendicular to the ventricular septum in parasternal short axis view [13] (Fig. 1). In one report, end systolic LVEI of 1.16 best identified the presence of PH [14]. Two-dimensional systolic RV/LV ratio was also measured in the parasternal short axis view [13] (Fig. 1). RV/LV ratio increases above 1 with worsening RV dilation. We have previously published excellent intra- and inter-rater reproducibility for LVEI, RV/LV ratio, RV strain and other continuous echocardiographic measures of RV function [15–19].

Descriptive statistics and echocardiographic parameters were summarized using median (25%, 75%) for continuous measures and frequencies for categorical variables. Echocardiographic variables were compared between the FETO and control groups at baseline, prior to CDH repair, post CDH repair, and at last available using non-parametric Wilcoxon rank-sum test. A linear regression model with robust variance and adjustment for the clustered nature of the data was used to evaluate the difference in the change in echocardiographic variables from baseline to last available between the FETO and control groups. A product term of the dichotomous group (FETO vs. control) and time operationalized as a dichotomous variable (baseline vs. last) was included in the model to evaluate differential change in echocardiographic parameters over time. If the interaction term was not statistically significant at the 0.05 level, the models were recalculated without the interaction term to assess if the echocardiographic parameters changed over time and by group. RV/LV ratio and LVEI were adjusted for left ventricular end-systolic Z-score to account for elevation in parameters due to LV hypoplasia. Results with a p-value less than 0.05 were considered as statistically significant.

## Results

Ten FETO patients and 22 control patients met inclusion criteria. Demographic and clinical characteristics are described in Table 1. Fetal observed/expected lung-to-head ratio was lower in FETO patients compared to controls [median (25%, 75%): 21.7 (21.4, 25.6) vs. 24.9 (23.5, 27.7), p = 0.06] as was gestational age at delivery [35.1 (34.5, 36.2) vs. 39.2 (38.8, 39.6), p < 0.001]. Extracorporeal membrane oxygenation (ECMO) utilization and pulmonary hypertension medications at discharge were lower in the FETO group compared to control patients (ECMO: 30% vs. 64%; medications: 0% vs. 27%), although these findings did not reach statistical significance. Overall survival was 90% in the FETO group and 82% in the control group.

**Table 1:** Demographic and clinical characteristics

Variable	FETO (n = 10)	Control (n = 22)	p-value
Sex			0.265
Female	3 (30%)	12 (55%)	
Male	7 (70%)	10 (45%)	
Race and ethnicity			0.426
Non-Hispanic White	8 (80%)	11 (50%)	
Non-Hispanic Black	0 (0%)	3 (14%)	
Multi-Racial/Other	1 (10%)	2 (9%)	
Hispanic/Latino	1 (10%)	6 (27%)	
Initial ultrasound LHR O/E	21.7 (21.4, 25.6)	24.9 (23.5, 27.7)	0.061
Gestational age at delivery, weeks	35.1 (34.5, 36.2)	39.2 (38.8, 39.6)	< 0.001
ECMO	3 (30%)	14 (64%)	0.128
Pulmonary hypertension medications at discharge	0 (0%)	6 (27%)	0.142
Survival			
Alive	9 (90%)	18 (82%)	> 0.999
Deceased in NICU after CDH repair	1 (10%)	1 (4%)	
Deceased after NICU discharge	0 (0%)	3 (14%)	

Data are expressed as n (%) or median (interquartile range)

*FETO* fetal endoscopic tracheal occlusion, *LHR* lung-to-head ratio, *O/E* observed to expected, *ECMO* extracorporeal membrane oxygenation, *NICU* neonatal intensive care unit, *CDH* congenital diaphragmatic hernia

At first echocardiogram, both groups demonstrated RV systolic dysfunction measured by low TAPSEZ, RVFAC, and RV strain (Table 2). RV/LV ratio and LVEI were lower in FETO patients compared to control patients [RV/LV: 1.10 (1.03, 1.24) vs. 1.57 (1.26, 1.76), p = 0.01 and LVEI 1.50 (1.38, 1.57) vs 1.94 (1.56, 2.31), p = 0.01], reflecting less RV dilation and less ventricular septal displacement, respectively. LV hypoplasia measured by 2-dimensional M-mode Z-scores was less severe in FETO patients compared to control patients [LVIDDZ − 2.12 (− 3.07, − 1.41) vs. − 3.50 (− 4.48, − 2.72), p = 0.01 and LVIDSZ − 1.55 (− 2.56, 0.80) vs. − 3.93 (− 4.69, − 2.67), p = 0.01]. While LV shortening fraction was lower in FETO patients [31.0 (25.0, 36.0) vs. 44.5 (36.0, 48.7), p = 0.02], LV function measured by GLS was similar.

**Table 2:** Pre-operative echocardiographic parameters in FETO vs. control patients

	Baseline/first echo			Pre-operative		
	FETO	Control	p value	FETO	Control	p value
Age at echo, d	1 (0.0, 1.0)	1 (1.0, 1.0)	0.22	16.5 (14.5, 22.8)	14.5 (11.0, 18.8)	0.23
Presence of PDA, (n, %)	9 (90%)	22 (100%)	0.31	1 (10%)	13 (59%)	0.02
TAPSEZ	− 3.5 (− 4.7, − 2.7)	− 2.7 (− 4.8, − 1.1)	0.45	− 1.6 (− 2.4, − 0.9)	− 0.9 (− 2.9, 0.3)	0.75
RVFAC	28.0 (20.1, 29.2)	26.2 (22.5, 32.1)	0.83	34.4 (31.7, 42.6)	38.9 (34.2, 43.9)	0.49
RVFWS	16.7 (14.0, 18.2)	14.9 (13.3, 17.1)	0.59	20.9 (18.1, 23.8)	23.5 (17.7, 26.2)	0.91
RVGLS	12.9 (10.9, 15.2)	12.5 (10.5, 16.4)	0.87	17.6 (15.4, 22.0)	18.8 (16.2, 23.1)	0.69
RV/LV	1.10 (1.03, 1.24)	1.57 (1.26, 1.76)	0.01	0.84 (0.79, 1.16)	1.01 (0.81, 1.59)	0.44
LVEI	1.50 (1.38, 1.57)	1.94 (1.56, 2.31)	0.01	1.25 (1.14, 1.57)	1.41 (1.21, 1.66)	0.41
LVIDDZ	− 2.12 (− 3.07, − 1.41)	− 3.50 (− 4.48, − 2.72)	0.01	− 1.75 (− 2.54, − 1.34)	− 2.02 (− 4.54, − 1.55)	0.39
LVIDSZ	− 1.55 (− 2.56, 0.80)	− 3.93 (− 4.69, − 2.67)	0.01	− 1.60 (− 2.24, − 1.19)	− 2.76 (− 4.43, − 0.88)	0.44
LVSF	31.0 (25.0, 36.0)	44.5 (36.0, 48.7)	0.02	39.0 (36.8, 40.0)	45.0 (34.5, 49.5)	0.26
LVGLS	21.8 (11.2, 22.5)	16.1 (10.8, 20.5)	0.42	21.2 (19.8, 27.2)	20.9 (17.8, 21.9)	0.25

Data are expressed as n (%) or median (interquartile range)

*FETO* fetal endoscopic tracheal occlusion, *d* days, *PDA* patent ductus arteriosus, *TAPSEZ* tricuspid annular plane systolic excursion Z-score, *RV* right ventricular, *FAC* fractional area change, *FWS* free wall strain, *GLS* global longitudinal strain, *LV* left ventricular, *EI* eccentricity index, *LVIDDZ* left ventricular internal dimension in diastole Z-score, *LVIDSZ* left ventricular internal dimension in systole Z-score, *SF* shortening fraction

The directionality of the effects noted at baseline were similar in the immediate pre-operative period, but there were no statistically significant differences in RV size or ventricular function between the two groups.

In the post-operative period, FETO patients demonstrated better RV function compared to control patients as measured by RVFAC [44.8 (42.8, 49.4) vs. 36.8 (29.4, 39.9), p < 0.01], RVGLS [24.8 (19.9, 26.6) vs. 19.5 (17.3, 21.2), p = 0.02], and RVFWS [28.5 (22.5, 31.5) vs. 23.3 (18.7, 26.3), p = 0.05] (Table 3). However, at last available echocardiogram there were no longer any differences in RV size or ventricular function between the two groups.

**Table 3:** Post-operative echocardiographic parameters in FETO vs. control patients

	Post-operative			Last available		
	FETO	Control	p value	FETO	Control	p value
Age at echo, *d*	30.0 (25.5, 41.5)	23.5 (17.5, 27.8)	0.02	122 (85.2, 142)	92.5 (46.0, 252)	0.94
Presence of PDA, n (%)	1 (10%)	10 (46%)	0.11	0 (0%)	4 (18%)	0.28
TAPSEZ	− 0.7 (− 1.9, 0.3)	− 1.4 (− 3.5, 0.8)	0.51	0.4 (− 1.7, 1.1)	0.1 (− 1.0, 1.3)	0.67
RVFAC	44.8 (42.8, 49.4)	36.8 (29.4, 39.9)	< 0.01	45.2 (40.5, 51.1)	45.1 (40.0, 48.2)	0.69
RVFWS	28.5 [22.5, 31.5)	23.3 (18.7, 26.3)	0.05	27.8 (24.6, 29.0)	24.2 (21.8, 28.1)	0.47
RVGLS	24.8 (19.9, 26.6)	19.5 (17.3, 21.2)	0.02	25.1 (20.9, 26.8)	22.3 (21.3, 25.7)	0.46
RV/LV	0.69 (0.60, 1.00)	0.93 (0.70, 1.10)	0.18	0.66 (0.57, 0.78)	0.65 (0.58, 1.16)	0.64
LVEI	1.11 (1.08, 1.17)	1.29 (1.11, 1.45)	0.09	1.01 (0.95, 1.04)	1.06 (0.99, 1.22)	0.14
LVIDDZ	− 1.05 (− 2.75, − 0.36)	− 1.76 (− 3.01, − 0.89)	0.46	− 1.98 (− 2.60, − 0.58)	− 0.43 (− 1.66, 0.31)	0.10
LVIDSZ	− 1.61 (− 2.44, − 0.55)	− 1.88 (− 3.34, − 0.49)	0.60	− 1.76 (− 2.42, − 0.69)	− 1.28 (− 1.96, 0.24)	0.40
LVSF	39.0 (37.0, 43.0)	41.0 (35.0, 45.0)	0.70	38.0 (36.3, 40.8)	40.0 (34.0, 46.0)	0.80
LVGLS	22.7 (21.6, 24.2)	21.2 (18.9, 23.8)	0.16	20.5 (19.1, 22.3)	21.9 (19.0, 23.2)	0.62

Data are expressed as n (%) or median (interquartile range)

*FETO* fetal endoscopic tracheal occlusion, *d* days, *PDA* patent ductus arteriosus, *TAPSEZ* tricuspid annular plane systolic excursion Z-score, *RV* right ventricular, *FAC* fractional area change, *FWS* free wall strain, *GLS* global longitudinal strain, *LV* left ventricular, *EI* eccentricity index, *LVIDDZ* left ventricular internal dimension in diastole Z-score, *LVIDSZ* left ventricular internal dimension in systole Z-score, *SF* shortening fraction

**Fig. 2:**
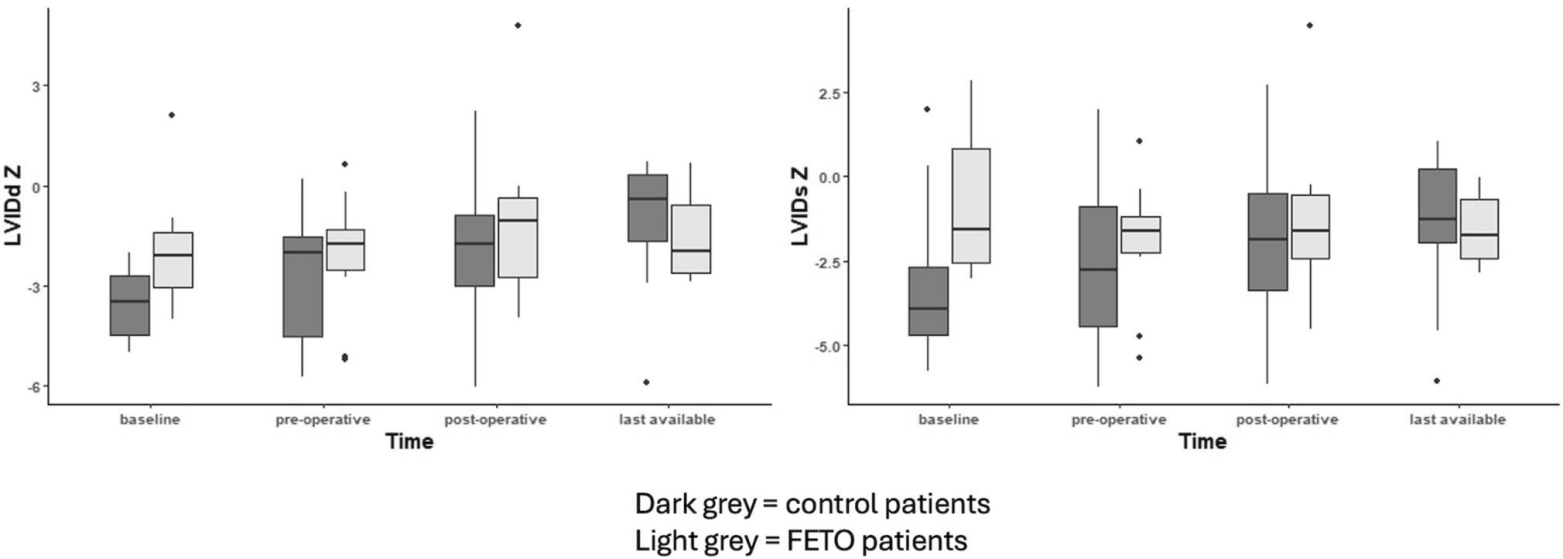
Changes in LV Z-scores for FETO and control patients. At last available echocardiogram, the increases in LVIDDZ and LVIDSZ were on average 2.22 and 2.75 units smaller, respectively, in the FETO patients than the control patients

**Fig. 3:**
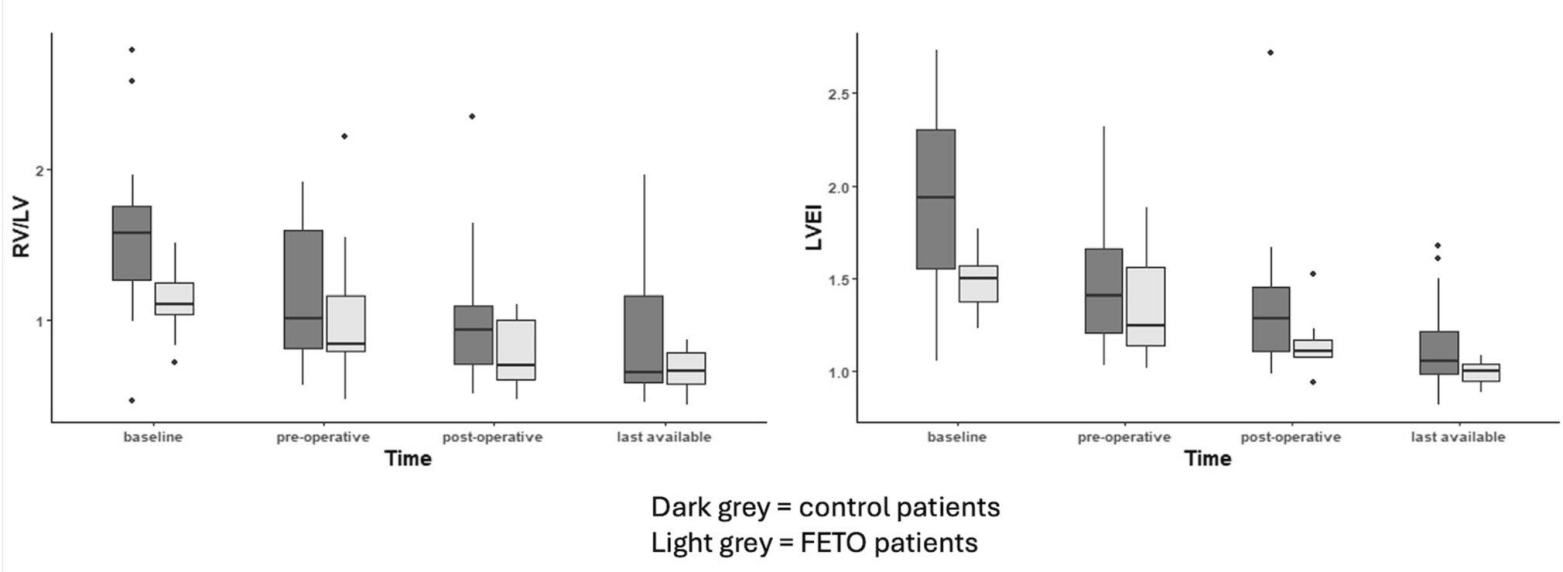
Changes in RV/LV ratio and LVEI for FETO and control patients. RV/LV ratio and LVEI decreased over time from first to last echocardiogram, and the decrease was greater in the FETO group on average

Changes in ventricular size and function from first to last echocardiogram differed between the groups. FETO patients demonstrated smaller increases in LV size measured by LVIDDZ and LVIDSZ compared to control patients (Table 4, Fig. 2). At last available echocardiogram, the increases in LVIDDZ and LVIDSZ from baseline were on average 2.22 and 2.75 units smaller, respectively, in the FETO patients than the control patients. RV/LV ratio and LVEI (adjusted for LV size) improved over time in both groups, but the improvements were greater in the FETO group on average (Table 4, Fig. 3). Improvements in RV function by TAPSEZ, RVFAC, RVFWS, and RVGLS were similar between the groups.

**Table 4:** Regression models measuring change in echocardiographic parameters from baseline to last available in FETO vs. controls

(A) Variable	TAPSEZ	RVFAC	RVFWS	RVGLS	RV/LV^a^	LVEI	LVIDDZ	LVIDSZ	LVSF	LVGLS
Intercept	− 2.8 (− 3.9, − 1.8)	26.4 (22.8, 29.9)	15.4 (13.2, 17.7)	13.1 (11.0, 15.2)	0.91 (0.70, 1.13)	1.49 (1.30, 1.65)	− 3.54 (− 4.09, − 2.99)	− 3.35 (− 4.50, − 2.20)	41.0 (32.4, 49.6)	15.4 (12.4, 18.5)
Visit: Last	3.1 (1.7, 4.5)	17.6 (13.7, 21.6)	10.1 (7.4, 12.8)	10.2 (7.5, 12.9)	− 0.25 (− 0.48, − 0.01)	− 0.48 (− 0.66, − 0.29)	2.62 (1.93, 3.31)	2.05 (0.67, 3.42)	0.00 (− 9.8, 9.8)	5.4 (2.0, 8.8)
Group: FETO	− 0.7 (− 2.3, 1.0)	0.0 (− 8.7, 8.8)	1.1 (− 3.8, 6.1)	0.4 (− 4.2, 5.0)	0.08 (− 0.17, 0.32)	− 0.06 (− 0.26, 0.14)	1.55 (0.34, 2.77)	2.51 (0.66, 4.37)	− 11.6 (− 21.8, − 1.3)	2.1 (− 3.7, 7.8)
FETO/Last Interaction	0.3 (− 1.7, 2.4)	1.7 (− 7.1, 10.4)	− 0.1 (− 5.2, 5.1)	0.8 (− 4.5, 6.0)	− 0.35 (− 0.67, − 0.04)	− 0.11 (− 0.35, 0.14)	− 2.22 (− 3.77, − 0.67)	− 2.75 (− 4.98, − 0.51)	9.05 (− 2.6, 20.7)	− 2.2 (− 8.4, 3.9)

(B) Variable	TAPSEZ	RVFAC	RVFWS	RVGLS	RV/LV	LVEI	LVSF	LVGLS
Intercept	− 2.9 (− 3.8, − 2.0)	26.1 (22.7, 29.5)	15.4 (13.4, 17.5)	13.0 (11.1, 14.9)	1.53 (1.29, 1.78)	1.51 (1.37, 1.66)	39.0 (32.3, 45.8)	15.8 (13.0, 18.5)
Visit: Last	3.2 (2.1, 4.3)	18.1 (14.5, 21.8)	10.1 (7.8, 12.4)	10.5 (8.2, 12.8)	− 0.64 (− 0.90, − 0.38)	− 0.52 (− 0.65, − 0.39)	3.3 (− 3.6, 10.1)	4.8 (1.9, 7.6)
Group: FETO	− 0.5 (− 1.7, 0.6)	0.8 (− 5.3, 7.0)	1.1 (− 2.2, 4.4)	0.8 (− 2.1, 3.7)	− 0.34 (− 0.53, − 0.14)	− 0.12 (− 0.22, − 0.02)	− 6.6 (− 11.1, − 2.0)	1.0 (− 2.3, 4.4)

Models in (A) include an interaction term to evaluate the change in the variables over the groups, over time. Models in (B) do not include the interaction term as it was not significant in model (A) for that echocardiographic parameter

*FETO* fetal endoscopic tracheal occlusion, *TAPSEZ* tricuspid annular plane systolic excursion Z-score, *RV* right ventricular, *FAC* fractional area change, *FWS* free wall strain, *GLS* global longitudinal strain, *LV* left ventricular, *EI* eccentricity index, *LVIDDZ* left ventricular internal dimension in diastole Z-score, *LVIDSZ* left ventricular internal dimension in systole Z-score, *SF* shortening fraction

^a^RV/LV ratio and LVEI were adjusted for adjusted for LVIDSZ

## Discussion

This is the first study to describe quantitative changes in heart size and function in patients with severe CDH who underwent the FETO procedure. In this cohort, those who underwent FETO demonstrated less severe RV dilation, less ventricular septal displacement, and less severe LV hypoplasia on first echocardiogram compared to control patients of similar CDH severity. While RV function improved in both groups over time, improvement in RV dilation and septal position measured by LVEI was greater in FETO patients. However, FETO patients demonstrated smaller increases in LV dimensions from first to last echocardiogram compared to control patients. As these echo measures are predictive of adverse outcomes in other forms of pediatric PH, these preliminary findings support future study of the cardiac consequences of this fetal intervention for severe CDH.

PH and ventricular dysfunction are leading determinants of mortality in CDH. Elevated RV afterload results in RV dysfunction, RV dilation, and RV failure. In children with pulmonary arterial hypertension (distinct from CDH-related PH), quantitative measures of ventricular size and function are critical to risk assessment. For example, the RV/LV ratio encompasses both ventricular septal shift and RV dilation and correlates with invasive measures of PH. For every 0.1 unit increase in RV/LV ratio, there is 10% increased hazard of adverse PAH-related events [20]. In other studies, worse RV function by TAPSE, worse RV dilation measured by RV/LV ratio, and smaller LV dimensions are associated with death or lung transplant [7]. These quantitative measures of RV size and systolic function have now been applied in CDH-related PH. Our group has previously applied these measures and demonstrated improvement in TAPSEZ, RVFAC, and RV strain after recovery from hernia repair in CDH patients of varying severity [21]. In the current study, RV function improved over time in patients with repaired severe CDH, but greater improvements in RV/LV ratio and LVEI were seen in those who underwent FETO compared to those who did not. These preliminary findings support future study to determine whether tracheal occlusion and fetal lung growth result in improved pulmonary vascular development and RV hemodynamics.

The impact of LV hypoplasia and dysfunction on CDH outcomes is now recognized. In a multi-center study using site-specific categorical definitions of ventricular dysfunction, survival was 80% in those with normal biventricular function, 74% in those with predominantly RV dysfunction, 57% in those with predominantly LV dysfunction, and only 51% in those with biventricular dysfunction [6]. LV strain was worse (reflecting worse LV systolic function) at 48 h of life in CDH patients who experienced the composite outcome of ECMO or death [22]. LVs in CDH patients are also often hypoplastic in addition to dysfunctional. LV volumes are smaller in CDH patients compared to control neonates [23]. LV hypoplasia can result in LV diastolic dysfunction, left atrial hypertension, and pulmonary edema [24, 25], critical when considering pulmonary vasodilator therapy. We recently demonstrated that CDH patients with both left heart hypoplasia and LV dysfunction have the highest risk of mortality [26].

In the current study, patients who underwent FETO demonstrated worse LV function by M-mode shortening fraction on initial echocardiogram compared to severe CDH patients who did not undergo FETO. This difference did not persist as LV function by shortening fraction was similar at all other time points, and quantitative strain assessment was similar at baseline and in serial imaging. However, there were differences in LV size between the two groups. On first echo, patients who underwent FETO had less severe LV hypoplasia as indicated by larger (“less negative”) LV end-diastolic and end-systolic dimension Z-scores. Yet, patients with FETO demonstrated smaller increases in LV dimensions from first to last echocardiogram compared to control patients. In FETO patients, median LV end-diastolic dimension Z-score at last echocardiogram was only − 1.98, reflecting a marked shift in LV size. The reasons for this are not clear in this small study. One possible explanation is that patients were born more prematurely after FETO. Some premature infants with bronchopulmonary dysplasia demonstrate LV diastolic dysfunction [27], which may be a manifestation of hypoplasia. There is mounting evidence for LV chamber hypoplasia in formerly preterm adolescents and young adults, even those without bronchopulmonary dysplasia [28]. The FETO procedure is known to result in higher incidence of preterm premature rupture of membranes and preterm birth. Underdevelopment of the LV with subsequent cardiac compromise are important adverse consequences of the intervention that deserve additional investigation.

## Limitations

This study has important limitations. Primarily, the sample size is small. Therefore, although we identified expected differences in clinical variables between the FETO and control patients, we did not adjust for the variables in the regression analyses. Additionally, the cross-sectional design does not allow us to assess the effect of medical or surgical interventions (i.e. pulmonary vasodilators, ECMO, inotropic support, ventilation strategies) on cardiac function. It is not clear if the improvement in ventricular function in either group is due to the “natural history” of CDH or due to a specific therapy.

## Conclusions

FETO patients demonstrated improved RV dilation and ventricular septal position but smaller increases in LV size compared to control patients of similar CDH severity. The impact of premature birth on these findings is not clear. Larger studies should investigate these echocardiographic changes to further understand the effect of FETO on cardiac development and ventricular function in severe CDH.

## Data Availability

The data that support the findings of this study are not openly available due to reasons of sensitivity and are available from the corresponding author upon reasonable request. Data are located in controlled access data storage at Children's Hospital of Philadelphia.

## References

[1] Ameis D, Khoshgoo N, Keijzer R (2017) Abnormal lung development in congenital diaphragmatic hernia. Semin Pediatr Surg 26:123–128 28641748 10.1053/j.sempedsurg.2017.04.011

[2] Gupta VS, Harting MT, Lally PA et al (2023) Mortality in congenital diaphragmatic hernia: a multicenter registry study of over 5000 patients over 25 years. Ann Surg 277:520–527 34334632 10.1097/SLA.0000000000005113

[3] Deprest J et al (2004) Fetoscopic tracheal occlusion (FETO) for severe congenital diaphragmatic hernia: evolution of a technique and preliminary results. Ultrasound Obstet Gynecol 24:121–6 15287047 10.1002/uog.1711

[4] Jani JC, Nicolaides KH, Gratacós E et al (2009) Severe diaphragmatic hernia treated by fetal endoscopic tracheal occlusion. Ultrasound Obstet Gynecol 34:304–310 19658113 10.1002/uog.6450

[5] Deprest JA, Nicolaides KH, Benachi A et al (2021) Randomized trial of fetal surgery for severe left diaphragmatic hernia. N Engl J Med 385:107–118 34106556 10.1056/NEJMoa2027030 PMC7613453

[6] Patel N, Lally PA, Kipfmueller F et al (2019) Ventricular dysfunction is a critical determinant of mortality in congenital diaphragmatic hernia. Am J Respir Crit Care Med 200:1522–1530 31409095 10.1164/rccm.201904-0731OC

[7] Ploegstra MJ, Roofthooft MT, Douwes JM et al (2014) Echocardiography in pediatric pulmonary arterial hypertension: early study on assessing disease severity and predicting outcome. Circ Cardiovasc Imaging 8:e000878 25552488 10.1161/CIRCIMAGING.113.000878

[8] Abman SH, Hansmann G, Archer SL et al (2015) Pediatric pulmonary hypertension: guidelines from the American Heart Association and American Thoracic Society. Circulation 132:2037–2099 26534956 10.1161/CIR.0000000000000329

[9] Colan SD (2009) Normal echocardiographic values for cardiovascular structures. In: Lai WW, Cohen MS, Geva T, Mertens L (eds) Echocardiography in pediatric and congenital heart disease. Wiley-Blackwell, West Sussex, pp 765–785

[10] Levy PT, Sanchez Mejia AA, Machefsky A et al (2014) Normal ranges of right ventricular systolic and diastolic strain measures in children: a systematic review and meta-analysis. J Am Soc Echocardiogr 27:549–560 24582163 10.1016/j.echo.2014.01.015 PMC4031687

[11] Koestenberger M, Ravekes W, Everett AD et al (2009) Right ventricular function in infants, children and adolescents: reference values of the tricuspid annular plane systolic excursion (TAPSE) in 640 healthy patients and calculation of z score values. J Am Soc Echocardiogr 22:715–719 19423286 10.1016/j.echo.2009.03.026

[12] Koestenberger M, Nagel B, Ravekes W et al (2011) Systolic right ventricular function in preterm and term neonates: reference values of the tricuspid annular plane systolic excursion (TAPSE) in 258 patients and calculation of Z-score values. Neonatology 100:85–92 21273793 10.1159/000322006

[13] Koestenberger M, Friedberg MK, Nestaas E et al (2016) Transthoracic echocardiography in the evaluation of pediatric pulmonary hypertension and ventricular dysfunction. Pulm Circ 6:15–29 27162612 10.1086/685051 PMC4860554

[14] Burkett DA, Patel SS, Mertens L et al (2020) Relationship between left ventricular geometry and invasive hemodynamics in pediatric pulmonary hypertension. Circ Cardiovasc Imaging 13:e009825. 10.1161/CIRCIMAGING.119.009825 32408829 10.1161/CIRCIMAGING.119.009825 PMC7236425

[15] Hopper RK, Wang Y, DeMatteo V et al (2018) Right ventricular function mirrors clinical improvement with use of prostacyclin analogues in pediatric pulmonary hypertension. Pulm Circ 8(2):2045894018759247. 10.1177/2045894018759247 29480089 10.1177/2045894018759247 PMC5843105

[16] DiLorenzo MP, Elci OU, Wang Y et al (2018) Longitudinal changes in right ventricular function in tetralogy of Fallot in the initial years after surgical repair. J Am Soc Echocardiogr 31:816–821 29627138 10.1016/j.echo.2018.02.013 PMC6035101

[17] Himebauch AS, Yehya N, Wang Y et al (2018) Early right ventricular systolic dysfunction and pulmonary hypertension are associated with worse outcomes in pediatric acute respiratory distress syndrome. Crit Care Med 46:e1055–e1062 30095502 10.1097/CCM.0000000000003358 PMC6185756

[18] Himebauch AS, Yehya N, Wang Y et al (2020) New or persistent right ventricular systolic dysfunction is associated with worse outcomes in pediatric acute respiratory distress syndrome. Pediatr Crit Care Med 21:e121–e128 31851127 10.1097/PCC.0000000000002206 PMC11215761

[19] Avitabile CM, Zhang X, Ampah SB et al (2022) Factors associated with discontinuation of pulmonary vasodilator therapy in children with bronchopulmonary dysplasia-associated pulmonary hypertension. J Perinatol 42:1246–1254 35676536 10.1038/s41372-022-01421-6

[20] Jone PN, Hinzman J, Wagner BD et al (2014) Right ventricular to left ventricular diameter ratio at end-systole in evaluating outcomes in children with pulmonary hypertension. J Am Soc Echocardiogr 27:172–178 24325962 10.1016/j.echo.2013.10.014 PMC3922965

[21] Avitabile CM, Wang Y, Zhang X et al (2020) Right ventricular strain, brain natriuretic peptide, and mortality in congenital diaphragmatic hernia. Ann Am Thorac Soc 17:1431–1439 32730099 10.1513/AnnalsATS.201910-767OC PMC7640722

[22] Patel N, Massolo AC, Paria A et al (2018) Early postnatal ventricular dysfunction is associated with disease severity in patients with congenital diaphragmatic hernia. J Pediatr 203:400–407 30195555 10.1016/j.jpeds.2018.07.062

[23] Altit G, Bhombal S, Van Meurs K et al (2018) Diminished cardiac performance and left ventricular dimensions in neonates with congenital diaphragmatic hernia. Pediatr Cardiol 39:993–1000 29523920 10.1007/s00246-018-1850-7

[24] Kinsella JP, Steinhorn RH, Mullen MP et al (2018) The left ventricle in congenital diaphragmatic hernia: implications for the management of pulmonary hypertension. J Pediatr 197:17–22 29628412 10.1016/j.jpeds.2018.02.040

[25] Maia PD, Gien J, Kinsella JP et al (2022) Hemodynamic characterization of neonates with congenital diaphragmatic hernia-associated pulmonary hypertension by cardiac catheterization. J Pediatr. 10.1016/j.jpeds.2022.11.028 36463937 10.1016/j.jpeds.2022.11.028

[26] Fraga MV, Hedrick HL, Rintoul NE et al (2024) Congenital diaphragmatic hernia patients with left heart hypoplasia and left ventricular dysfunction have highest odds of mortality. J Pediatr 1:114061. 10.1016/j.jpeds.2024.114061 10.1016/j.jpeds.2024.114061 PMC11239301 38636784

[27] Mourani PM, Ivy DD, Rosenberg AA et al (2008) Left ventricular diastolic dysfunction in bronchopulmonary dysplasia. J Pediatr 152:291–293 18206706 10.1016/j.jpeds.2007.11.006 PMC2259289

[28] Goss KN, Haraldsdottir K, Beshish AG et al (2020) Association between preterm birth and arrested cardiac growth in adolescents and young adults. JAMA Cardiol 5:910–919 32432648 10.1001/jamacardio.2020.1511 PMC7240643

